# *Bupleurum chinense* Polysaccharide Improves LPS-Induced Senescence of RAW264.7 Cells by Regulating the NF-*κ*B Signaling Pathway

**DOI:** 10.1155/2020/7060812

**Published:** 2020-12-15

**Authors:** Mengran Xu, Shengyang Sun, Junhong Ge, Ye Shen, Tan Li, Xin Sun

**Affiliations:** ^1^College of Pharmacy, Jilin Medical University, Jilin, China; ^2^Jilin Provincial Laboratory of Molecular Geriatrics, Jilin, China; ^3^College of Medicine, Beihua University, Jilin, China

## Abstract

Macrophages are important inflammatory cells that play a vital role in inflamm-aging. *Bupleurum chinense* polysaccharide (BCP), an effective component of the *Bupleurum chinense* herb, exerts multiple beneficial pharmacological effects, such as improving immunity and antioxidant activity. However, the effects of BCP on macrophage-aging and inflamm-aging are yet to be established. In this study, we examined the effects of BCP on proliferation, inflammatory cytokines, *β*-galactosidase (SA-*β*-gal), senescence-associated heterochromatin foci (SAHF), reactive oxygen species (ROS), mitochondrial membrane potential, p53, p16, and p65/NF-*κ*B signaling proteins in lipopolysaccharide (LPS)-stimulated RAW264.7 cells. BCP significantly inhibited production of interleukin-1*α* (IL-1*α*), interleukin-6 (IL-6), and tumor necrosis factor-*α* (TNF-*α*), reduced the expression of SA-*β*-gal and formation of SAHF, as well as ROS level, and stabilized the mitochondrial membrane potential in RAW264.7 cells stimulated with LPS. Furthermore, BCP inhibited the expression of aging-related genes, p53 and p16, suppressed phosphorylation of p65 protein, and enhanced the expression of I-*κ*B*α* protein through the NF-*κ*B signaling pathway in LPS-stimulated RAW264.7 cells. Accordingly, we conclude that BCP effectively suppresses inflamm-aging by reducing inflammatory cytokine levels and oxidative stress production following activation of the NF-*κ*B signaling pathway in RAW264.7 cells stimulated with LPS. Our collective findings support the utility of BCP as a novel pharmaceutical agent with potential anti-inflamm-aging effects.

## 1. Introduction

The concept of inflamm-aging, referring to a state of chronic progressive increase in inflammatory response during the development of aging, was first proposed by the group of Franceschi in 2000 [1]. Inflamm-aging is an important contributory factor to the rate of aging and life span [2–4], which is also associated with several severe aging-related diseases, such as cardiovascular disorders, type 2 diabetes mellitus, and neurodegenerative diseases [5–7]. Several recent reports on the oxidative inflammation theory, cytokine theory, and DNA damage theory further support the concept of inflamm-aging [8, 9]. Oxidative stress, inflammation, and aging have a mutually causal relationship [10], and proinflammatory cytokines play a critical role in the inflamm-aging process. Continuous and low-intensity stimulated inflammation is unable to trigger stable anti-infection and tissue damage repair effects. Consequently, high levels of proinflammatory cytokines accumulate in the circulation that contribute to an inflammatory environment for tissues and organs, leading to oxidative stress and aging [11, 12]. Oxidative stress also results in aging and affects homeostasis and health of the body [13]. Macrophages, a common immune cell type originating from bone marrow precursor cells, are widely distributed in the body and play an important defensive role in innate and acquired immunity [14]. Activation of macrophages is regarded as the first line of defense against infections through phagocytizing pathogenic media, which prevents inflammation and tissue damage [15]. In addition, aging of macrophages is one of the primary causes of inflamm-aging. Therefore, exploration of the impact of drugs on inflamm-aging through macrophages is a significant concern for researchers [16].

Chinese herbal medicine has been traditionally used for over a century owing to several advantages including unique natural resources, fewer side-effects, extensive pharmacological effects, and multiple pharmaceutical ingredients [17, 18]. Polysaccharides are natural biological macromolecular compounds composed of monosaccharides. Due to their lack of side-effects on normal cells *in vivo*, polysaccharides are also known as “a biological reaction regulators” and represent an important active component of traditional Chinese medicines (TCMs) [19–21]. “*Bupleurum chinense* DC. (*Bupleurum* spp.), first included in the Chinese Pharmacopoeia in 1963, is a well-known traditional Chinese medicine used for more than a thousand years that contains polysaccharide as the main therapeutic component [22]. *Bupleurum chinense* polysaccharide (BCP) is reported to exert multiple pharmacological effects, such as antitumor activity, protection against liver injury, antipulmonary fibrosis, and regulation of immune system function [23–25]. Previous studies have shown that BCP has a significant *in vitro* antioxidant effect and delays H_2_O_2_-induced aging of lung epithelial cells in mice [26]. However, to our knowledge, no reports have explored the effects of BCP on macrophage-aging and inflamm-aging. In this study, lipopolysaccharide (LPS) was used to stimulate mouse macrophage RAW264.7 leukemia cells to generate a macrophage-inflamm-aging model. We determined the effects of BCP on cell viability via the cell proliferation assay, senescence-associated secretory phenotype (SASF) associated with inflammatory cytokines via ELISA, senescence-related *β*-galactosidase (SA-*β*-gal) changes using SA-*β*-gal staining, senescence-associated heterochromatin foci (SAHF), reactive oxygen species (ROS), and mitochondrial membrane potential with the aid of fluorescence microscopy, and expression of senescence-related markers, such as p16 and p53 via western blot. Finally, the NF-*κ*B signaling pathway was taken as the target to explore the related mechanisms. Our findings provide a theoretical basis for further research on the anti-inflamm-aging effect of BCP.

## 2. Materials and Methods

### 2.1. Materials

BCP (*Bupleurum chinense* polysaccharide) was prepared at the Life Science Center, Beihua University. In brief, the extraction method was as follows: the crude drug was extracted using water and alcohol precipitation, fractionated, dialyzed, lyophilized, and eluted with an ÄKTA Explore 100 FPLC purification system (Amersham Biosciences Division of GE Healthcare) to obtain purified BCP. The total carbohydrate content of purified BCP was 97.5%. BCP was mainly composed of arabinose (Ara), galactose (Gal) and glucose (Glc) with a molar mass ratio of 2.1 : 2.5 : 1 [27, 28].

Antibodies specific for p65, p53, I-*κ*B*α*, p16 and their phosphorylated counterparts, and *β*-actin, and LPS (biological source: *Escherichia coli*, O111, B4) were purchased from Sigma Company (St. Louis, Missouri, USA), RPMI1640 medium and fetal bovine serum from Gibco Company (California, USA), enzyme linked immunosorbent assay (ELISA) kits were purchased from ABclonal Company (Wuhan, China), and MTT, NP40 protein lysis buffer, penicillin, streptomycin, BCA protein assay, cell senescence *β*-galactosidase staining, reactive oxygen species assay, and mitochondrial membrane potential assay (with JC-1 dye) kits were acquired from Biyuntian Company (Shanghai, China).

### 2.2. Methods

#### 2.2.1. Cell Culture

RAW264.7 mouse macrophage leukemia cells (a mouse peritoneal macrophage cell line) purchased from Beijing Dingguo Changsheng Biotechnology Co., Ltd. (Beijing, China) were cultured in RPMI1640 containing 10% heat-inactivated fetal bovine serum (FBS) and 1% penicillin-streptomycin at 37°C in a 5% CO_2_ incubator.

#### 2.2.2. MTT Cell Proliferation Assay

Cell proliferation was measured via the MTT assay. RAW264.7 cells (5 × 10^3^ cells/well) were seeded in an RPMI 1640 medium containing 10% FBS and 1% penicillin-streptomycin in a 96-well plate and cultured at 37°C in a 5% CO_2_ incubator overnight. Firstly, we established the potential toxic effects on RAW264.7 cells after treatment with different concentrations of BCP. The blank group (medium only), control group (cells with medium), and BCP groups (cells treated with different BCP concentrations) were incubated for 24 h. An aliquot of medium (200 *μ*L) and MTT solution (20 *μ*L) were added into the wells (at a final concentration of 0.5 mg/mL), followed by incubation at 37°C for 4 h. After discarding of the medium, 150 *μ*L of DMSO was added into the wells. Absorbance at 490 nm was measured with a microplate reader, and cell proliferation was calculated according to the following formula.

The effect of BCP on proliferation was observed after using LPS to induce the inflammatory response of RAW264.7 cells. The following groups were examined: blank group (medium only), control group (cells with medium), LPS group (cells stimulated with 1 *μ*g/mL LPS for 24 h), and BCP groups (cells pretreated with different BCP concentrations for 24 h followed by 24 h stimulation with LPS). Next, 200 *μ*L medium and 20 *μ*L MTT solution were added to the wells (at a final concentration of 0.5 mg/mL) and incubated at 37°C for 4 h. Subsequently, the medium was discarded and 150 *μ*L DMSO was added to the wells. Absorbance at 490 nm was measured using a microplate reader, and cell proliferation was calculated according to the following formula:

(1) $$\begin{array}{c} \text{cell proliferation }\%=\frac{A_{\text{test group}}-A_{\text{blank group}}}{A_{\text{control group}}-A_{\text{blank group}}}\times 100\%, \end{array}$$

whereby *A*_test_ represents absorbance measured in drug-treated groups (LPS group or BCP groups), *A*_con_ represents absorbance measured in the control group without drugs, and *A*_blan_ represents absorbance measured in the blank group without cells and drugs.

#### 2.2.3. Detection of Inflammatory Cytokines

Expression of inflammatory cytokines in LPS-stimulated cells was detected using ELISA. RAW264.7 cells (6 × 10^4^ cells/well) were seeded in an RPMI 1640 medium containing 10% FBS and 1% penicillin-streptomycin in a 6-well plate and cultured in a 5% CO_2_ incubator at 37°C overnight. Cells in the control group were not treated with drugs, those in the LPS group were stimulated with LPS for 24 h, and those in the remaining groups were pretreated with BCP (50, 100, and 200 *μ*g/mL) for 24 h followed by LPS (1 *μ*g/mL) for 24 h. Cell supernatant fractions were collected, and expression levels of the inflammatory cytokines interleukin-1*α* (IL-1*α*), interleukin-6 (IL-6), and tumor necrosis factor-*α* (TNF-*α*) were detected via ELISA.

#### 2.2.4. ROS Detection and Analysis

ROS produced after LPS stimulation in cells were detected using a specific reactive oxygen species assay kit. RAW264.7 cells (6 × 10^4^ cells/well) were seeded in an RPMI 1640 medium containing 10% FBS and 1% penicillin-streptomycin in a 6-well plate and cultured in a 5% CO_2_ incubator at 37°C overnight. Cells in the control group were not treated with drugs, the LPS group was stimulated with LPS for 24 h, and the BCP (200 *μ*g/mL) group was pretreated with BCP for 24 h followed by LPS stimulation (1 *μ*g/mL) for 24 h. The DCFH-DA fluorescent probe prepared with a serum-free medium (10 *μ*mol/mL) was added to cover the cell surface, incubated at 37°C for 20 min, and washed three times with serum-free medium. Staining was observed under an inverted fluorescence microscope (Olympus, Tokyo, Japan). ImageJ image-processing software used to analyze fluorescence images and fluorescence intensity of ROS staining calculated in three random fields of cells in each group.

#### 2.2.5. Analysis of Mitochondrial Membrane Potential

Changes in the mitochondrial membrane potential after LPS stimulation were detected using a JC-1 staining kit. JC-1 is an ideal fluorescent-labeling probe widely used to detect mitochondrial membrane potential (∆Ψm). At high mitochondrial membrane potential, JC-1 accumulates in the matrix of mitochondria to form polymers displaying red fluorescence. RAW264.7 cells (6 × 10^4^ cells/well) were seeded in the RPMI 1640 medium containing 10% FBS and 1% penicillin-streptomycin in a 6-well plate and cultured at 37°C in a 5% CO_2_ incubator overnight. Cells in the control group were not treated with drugs, the LPS group was stimulated with LPS for 24 h, and the BCP (200 *μ*g/mL) group was pretreated with BCP for 24 h followed by LPS (1 *μ*g/mL) for 24 h. Treated cells were washed with PBS, followed by addition of a 1 mL culture medium and 1 mL JC-1 dye working solution into each well in 6-well plates. After vigorous mixing, cells were incubated at 37°C for 20 min. The supernatant was discarded, 1 mL JC-1 staining buffer was added to each well, and cells were washed three times. After discarding the supernatant, a 2 mL cell culture medium was added into each well. Staining was observed under an inverted fluorescence microscope, and ImageJ image-processing software was used to analyze fluorescence images and calculate the fluorescence intensity of mitochondrial membrane potential staining in three random fields of cells in each group.

#### 2.2.6. SA-*β*-Gal Staining Analysis

Cellular SA-*β*-gal levels were measured after stimulation LPS using a cell senescence *β*-galactosidase staining kit. RAW264.7 cells (6 × 10^4^ cells/well) were seeded in the RPMI 1640 medium containing 10% FBS and 1% penicillin-streptomycin in a 6-well plate and cultured at 37°C in a 5% CO_2_ incubator overnight. Cells in the control group were not treated with drugs, the LPS group was stimulated with LPS for 24 h, and the BCP (200 *μ*g/mL) group was initially pretreated with BCP for 24 h followed by LPS stimulation (1 *μ*g/mL) for 24 h. Treated cells were washed twice with PBS, incubated with fixation fluid at room temperature for 15 min, and further washed three times with PBS for 3 min each time. According to the manufacturer's instructions, cells were stained and incubated at 37°C without CO_2_ overnight. Stained cells were examined under an inverted fluorescence microscope.

#### 2.2.7. SAHF Staining Analysis

DAPI staining was used to detect the occurrence of SAHF. RAW264.7 cells (6 × 10^4^ cells/well) were seeded in the RPMI 1640 medium containing 10% FBS and 1% penicillin-streptomycin in a 6-well plate and cultured at 37°C in a 5% CO_2_ incubator overnight. Cells in the control group were not treated with drugs, the LPS group was stimulated with LPS for 24 h, and the BCP (200 *μ*g/mL) group was pretreated with BCP for 24 h followed by stimulation with LPS (1 *μ*g/mL) for 24 h. After treatment, the supernatant was aspirated, and cells were fixed with fixation fluid for 5 min, washed with PBS three times. DAPI staining solution was added into 6-well plates to cover the cell surface. After incubation at 37°C for 5 min, cells were washed with PBS three times. Staining was observed under an inverted fluorescence microscope, and ImageJ image-processing software was used to analyze fluorescence images and calculate fluorescence intensity of DAPI staining in three random fields of cells in each group.

#### 2.2.8. Western Blot Analysis

Protein extracted from RAW264.7 cells was prepared with NP40 protein lysis buffer according to the manufacturer's instructions. The total protein concentrations in the extracts were determined with the BCA protein assay. Proteins were separated via SDS-PAGE (12%), electrotransferred onto polyvinylidene fluoride (PVDF) membranes, blocked with blocking buffer containing 5% (w/v) skimmed milk, and incubated in PBST (PBS with 0.1% w/v Tween-20) containing primary antibodies specific for p65, p53, I-*κ*B*α*, p16 proteins, phosphorylated p65, p53, I-*κ*B*α*, p16 proteins, and *β*-actin at 4°C overnight. PVDF membranes were washed three times with PBST for 10 min each time and incubated with horseradish peroxidase for 1 h. Membranes were subsequently washed in the same way, and the hypersensitive ECL chemiluminescence kit was used for development after aspiration of liquid. Protein bands were quantified based on the mean ratio of the normalized integral optical density of *β*-actin or the protein.

#### 2.2.9. Statistical Analysis

All experiments were repeated three times, and data were expressed as means ± SD. The results were analyzed using GraphPad Prism 6. Differences were considered statistically significant at *P* < 0.05.

## 3. Results

### 3.1. Effect of BCP on Proliferation of LPS-Stimulated Macrophages

**Figure 1:**
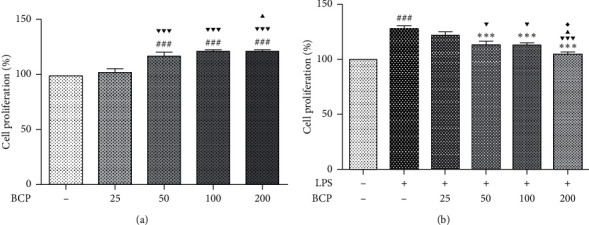
Effects of BCP on proliferation of RAW264.7 macrophages with and without LPS stimulation. Results are presented as means ± SD. ^###^*P* < 0.001, compared with the control group; ^*∗∗∗*^*P* < 0.001, compared with the LPS model group; ^▼▼▼^*P* < 0.001, ^▼^*P* < 0.05, compared with the 25 *μ*g/mL BCP group; ^▲^*P* < 0.05, compared with the 50 *μ*g/mL BCP group; ^◆^*P* < 0.05, compared with the 100 *μ*g/mL BCP group.

The MTT assay was employed to detect the toxicity of BCP on RAW264.7 cells. Proliferation of macrophages stimulated with LPS (1 *μ*g/mL) or/and treated with BCP (25, 50, 100, and 200 *μ*g/mL) for 24 h was assessed. As shown in Figure 1(a), compared with the control group (BCP−), proliferation of macrophages in groups treated with different concentrations of BCP was increased. Our data suggest that BCP within a concentration range of 50–200 *μ*g/mL promotes proliferation of normal RAW264.7 cells (*P* < 0.001) without causing toxicity to the cells themselves.

As shown in Figure 1(b), compared with the control group (LPS−, BCP−), proliferation of macrophages in the LPS group (LPS 1 *μ*g/mL+, BCP−) was significantly increased, reaching 127.76 ± 2.44% (*P* < 0.001). Compared with the LPS group, proliferation of macrophages was inhibited in the BCP treatment groups in a concentration-dependent manner, in particular, reduced to 104.88 ± 2.02% in the BCP treatment group (LPS 1 *μ*g/mL+, BCP 200 *μ*g/mL+; *P* < 0.001). LPS could be used to induce inflammation of macrophages. Based on these results, we propose that the inflammatory response induced by LPS stimulates abnormal proliferation of RAW264.7 macrophages, which can be effectively inhibited by BCP without causing toxicity.

### 3.2. BCP Inhibits LPS-Stimulated Production of Inflammatory Cytokines in Macrophages

Since proinflammatory cytokines are closely related to inflamm-aging, cytokine production by macrophages after treatment with LPS and BCP was examined.

**Figure 2:**
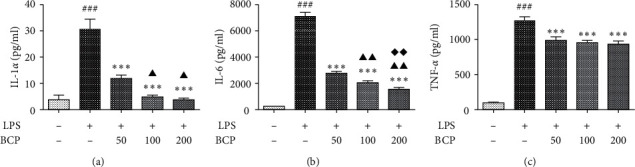
Effects of BCP on LPS-stimulated inflammatory cytokines. (a) IL-1*α*; (b) IL-6; (c) TNF-*α*. Results are presented as means ± SD. ^###^*P* < 0.001, compared with the control group; ^*∗∗∗*^*P* < 0.001, compared with the LPS group; ^▲▲^*P* < 0.01, ^▲^*P* < 0.05, compared with the BCP (50 *μ*g/mL) group; ^◆◆^*P* < 0.01, compared with the BCP (100 *μ*g/mL) group.

As shown in Figure 2(a), the IL-1*α* content was low in the control group (LPS−, BCP−), determined as 3.61 ± 1.86 pg/mL. The IL-1*α* content in the LPS group (LPS 1 *μ*g/mL+, BCP−) was significantly higher than that in the control group (30.81 ± 3.60 pg/mL; *P* < 0.001). The IL-1*α* content of groups treated with BCP was decreased relative to that in the LPS group in a concentration-dependent manner (*P* < 0.001). IL-1*α* levels in the 100 and 200 *μ*g/mL BCP groups were significantly lower than that in the 50 *μ*g/mL BCP group and, in particular, decreased to 3.64 ± 0.62 pg/mL in the 200 *μ*g/mL BCP treatment group.

As shown in Figure 2(b), the IL-6 content in the control group (LPS−, BCP−) was 230.33 ± 11 pg/mL. The IL-6 content in the LPS group (LPS 1 *μ*g/mL+, BCP-) was significantly higher than that in the control group (7157.56 ± 281.65 pg/mL; *P* < 0.001). After treatment with different concentrations of BCP, IL-6 expression was decreased in a concentration-dependent manner, with the most significant difference (1581.11 ± 94.82 pg/mL) in the 200 *μ*g/mL treatment group.

As shown in Figure 2(c), the TNF-*α* content in the control group (LPS−, BCP−) was 97.99 ± 3.66 pg/mL. The TNF-*α* content in the LPS group (LPS 1 *μ*g/mL+, BCP−) was significantly higher than that in the control group (1276.7 ± 42.68 pg/mL; *P* < 0.001). The TNF-*α* contents in the groups treated with BCP different concentrations were lower than that in the LPS model group (*P* < 0.001), with the lowest level recorded in the 200 *μ*g/mL BCP group (940.19 ± 43.02 pg/mL).

Our results indicate that BCP significantly inhibits the expression of inflammatory cytokines after stimulation with LPS.

### 3.3. Effects of BCP on ROS and Mitochondrial Membrane Potential of LPS-Stimulated Macrophages

**Figure 3:**
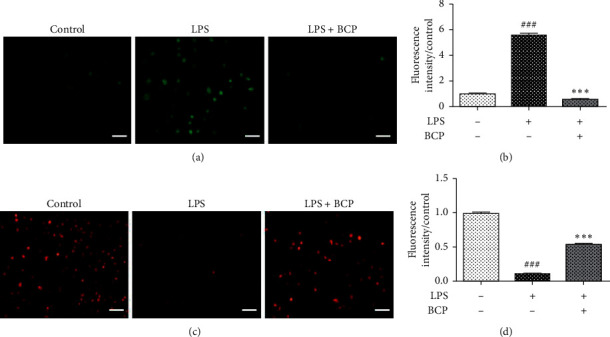
Effects of BCP on ROS and mitochondrial membrane potential of LPS-stimulated macrophages. (a) ROS detection; (b) ROS fluorescence intensity analysis; (c) mitochondrial membrane potential detection; (d) fluorescence intensity analysis of mitochondrial membrane potential. Results are presented as means ± SD. ^###^*P* < 0.001, compared with the control group, ^*∗∗∗*^*P* < 0.001, compared with the LPS model group.

In view of the finding that ROS are normal metabolites of various redox reactions in cells [29], ROS levels in LPS-induced macrophages were determined. To this end, ROS staining and fluorescence intensity analysis of cells in each group was performed (Figures 3(a) and 3(b)). The control group (LPS−, BCP−) contained fewer fluorescence-stained cells with lower intensity. Compared with the control group, green fluorescence-stained cells in the LPS group (LPS 1 *μ*g/mL+, BCP−) were more evident, and both ROS expression and fluorescence intensity were increased (*P* < 0.001). Compared with the LPS group, the proportion of green fluorescence-stained cells in the BCP group (LPS 1 *μ*g/mL+, BCP 200 *μ*g/mL+) was significantly reduced, along with fluorescence intensity (*P* < 0.001). The results indicate that BCP reduces ROS production of LPS-stimulated macrophages.

The JC-1 staining kit was used to detect changes in mitochondrial membrane potential in LPS-stimulated cells, whereby red fluorescence was observed when the mitochondrial membrane potential was at a normal level, which decreased with reduction of mitochondrial membrane potential. Staining of mitochondrial membrane potential and analysis of fluorescence intensity are presented in Figures 3(c) and 3(d). The control group (LPS−, BCP−) contained numerous red fluorescent-stained cells with high fluorescence intensity, signifying normal mitochondrial membrane potential. Compared with the control group, the number of red fluorescent-stained cells and fluorescence intensity were lower and mitochondrial membrane potential began to decline in the LPS group (LPS 1 *μ*g/mL+, BCP−; *P* < 0.001). After BCP (200 *μ*g/mL) treatment, fluorescent-stained cell number and fluorescence intensity increased were significantly increased relative to the LPS group (*P* < 0.001), indicating that BCP stabilizes the mitochondrial membrane potential of LPS-stimulated macrophages.

### 3.4. Effect of BCP on the Macrophage Aging Induced by LPS

**Figure 4:**
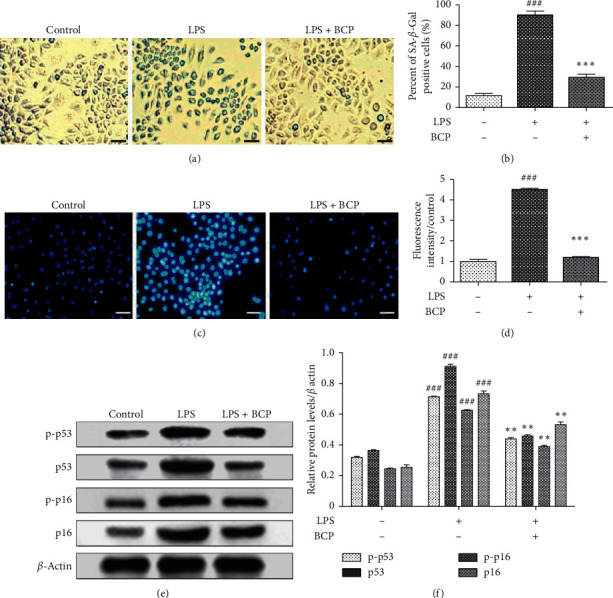
Effects of BCP on macrophage-aging induced by LPS. (a) SA-*β*-gal staining; (b) SA-*β*-gal-positive cell count analysis; (c) DAPI staining; (d) DAPI fluorescence intensity analysis; (e) electrophoresis images of p53, p16, phosphorylated p53 and p16, and *β*-actin protein; (f) p-p53/*β*-actin, p53/*β*-actin, p-p16/*β*-actin, and p16/*β*-actin column charts. Results are presented as means ± SD. ^###^*P* < 0.001, compared with the control group, ^*∗∗∗*^*P* < 0.001, ^*∗∗*^*P* < 0.01, compared with the LPS model group.

SA-*β*-gal was the first molecular marker used to specifically identify aging cells [30]. Abundant staining of cells and upregulation of SA-*β*-gal were observed in the LPS group (LPS 1 *μ*g/mL+, BCP−) while the number of blue-stained cells was decreased in the BCP treatment group (Figure 4(a)). As shown in Figure 4(b) (SA-*β*-gal-positive cell count analysis), the SA-*β*-gal blue-stained cell content in the LPS group was significantly higher than that in the control group (LPS−, BCP−; *P* < 0.001) while the number of stained cells in the BCP group (LPS 1 *μ*g/mL+, BCP 200 *μ*g/mL+) was lower than that in the LPS group (*P* < 0.001). Based on the results, we suggest that LPS-stimulated inflammation occurs concomitantly with progression of aging and BCP reduces expression of SA-*β*-gal in LPS-induced macrophages.

Senescence-associated heterochromatin foci (SAHF), an important indicator in the process of senescence, refers to the heterochromatin structure of point-like foci in nuclei of senescent cells. As shown in Figures 4(c) and 4(d), low levels of SAHF were detected in the control group (LPS−, BCP−). Compared with the control group, the number of fluorescent-stained cells and fluorescence intensity increased significantly and SAHF were more prevalent in the LPS group (LPS 1 *μ*g/mL+, BCP−; *P* < 0.001). After BCP (200 *μ*g/mL) treatment, both the SAHF level and fluorescence intensity were decreased (*P* < 0.001), clearly indicating that BCP reduces the occurrence of SAHF in LPS-induced macrophages.

Based on the finding that inflammation promotes the aging process, expression levels of p53 and p16 as well as their phosphorylated proteins in classic age-associated genes were further detected via western blot. As shown in Figure 4(e), levels of p53 and p16 proteins and phosphorylated p53 and p16 proteins in the LPS group (LPS 1 *μ*g/mL+, BCP−) were significantly higher than those in the control group (LPS−, BCP−; *P* < 0.001) while levels in the BCP group (LPS 1 *μ*g/mL+, BCP 200 *μ*g/mL+) were lower relative to the LPS group (*P* < 0.01). The grayscale analysis of Figure 4(e) is presented in Figure 4(f).

### 3.5. Effects of BCP on Expression of Proteins Related to the NF-*κ*B Signaling Pathway of LPS-Stimulated Macrophages

**Figure 5:**
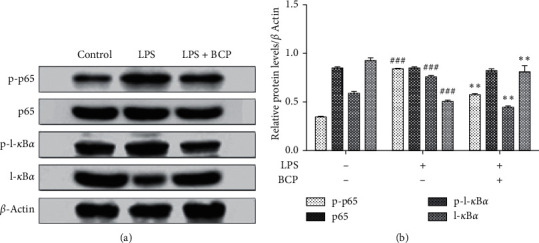
Effects of BCP on proteins related to the NF-*κ*B signaling pathway of LPS-stimulated macrophages. (a) Electrophoresis images of p65, I-*κ*B*α*, phosphorylated p65 and I-*κ*B*α*, and *β*-actin proteins; (b) p-p65/*β*-actin, p65/*β*-actin, p-I-*κ*B*α*/*β*-actin, and I-*κ*B*α*/*β*-actin column charts. Results are presented as means ± SD. ^###^*P* < 0.001, compared with the control group, ^*∗∗*^*P* < 0.01, compared with the LPS model group.

To explore whether the molecular mechanism by which BCP inhibits inflamm-aging is related to the NF-*κ*B pathway, expression levels of p65 and I-*κ*B*α* proteins and their phosphorylated proteins in the NF-*κ*B signaling pathway were detected via western blot in cells. As shown in Figure 5(a), compared with the control group (LPS−, BCP−), expression of I-*κ*B*α* protein was decreased and that of phosphorylated p65 and I-*κ*B*α* proteins increased in the LPS group (LPS 1 *μ*g/mL+, BCP−; *P* < 0.001). Compared with the LPS group, expression of I-*κ*B*α* protein increased, and that of phosphorylated p65 and I-*κ*B*α* proteins decreased in the BCP treatment group (LPS 1 *μ*g/mL+, BCP 200 *μ*g/mL+) to a significant extent (*P* < 0.01). The grayscale analysis of Figure 5(a) is depicted in Figure 5(b).

## 4. Discussion

Oxidative stress-inflammation-aging theory is an important theory in the pathogenesis of inflamm-aging [31]. During the inflamm-aging process, free radicals, such as ROS, which accumulate in macrophages and other inflammatory cells, induce oxidative stress and release of multiple proinflammatory cytokines that cause a systemic chronic inflammatory response in the body. The inflammatory cytokines further strengthen the oxidative stress response and weaken the antioxidant capacity of cells, resulting in damage to cellular protein structure and functions as well as DNA mutation, eventually leading to aging and death [32–34]. LPS, an endotoxin released by Gram-negative bacteria, exerts an immunomodulatory effect. Immune cells express a variety of mitogen receptor glycosyl molecules on their surface and continually activate a proliferative response due to mitogen stimulations. Thus, LPS as a mitogen can activate macrophages that are polarized into M1 type [35] to release several inflammatory cytokines [36]. In one way, these cytokines recruit macrophages and promote their proliferation to enhance local anti-infection ability. However, with increasing macrophage proliferation, more inflammatory cytokines are released, intensifying the inflammation process. In another way, these cytokines also participate in intracellular inflammation to remove related pathogens as an immunomodulatory role. These effects further trigger a burst of ROS while regulating the immune system, resulting in inflamm-aging [37–39]. Our initial experiments showed that BCP promotes proliferation of normal RAW264.7 cells, indicating no toxic effects on normal macrophages. LPS is commonly used for stimulation of mouse RAW264.7 macrophages and enhancing their proliferation [40–43]. Interestingly, while RAW 264.7 cell proliferation was promoted by LPS stimulation, preaddition of 50–200 *μ*g/mL BCP significantly inhibited proliferation in a dose-dependent manner. These results suggest that BCP effectively suppresses proliferation of LPS-induced macrophages through exerting an anti-inflammatory effect. Besides, levels of proinflammatory cytokines such as IL-1*α*, IL-6, and TNF-*α* were significantly increased in LPS-induced RAW264.7 cells. Upon treatment with 50, 100, and 200 *μ*g/mL BCP, release of inflammatory cytokines was inhibited in a concentration-dependent manner. Since BCP at 200 *μ*g/mL exerted good inhibitory effects against both proliferation and release of inflammatory cytokines in RAW264.7 cells, this concentration was selected as the optimal dose to further investigate the anti-inflammatory and antiaging effects of BCP as well as related mechanisms.

Inflammatory cytokines are closely related to the nuclear factor kappa B (NF-*κ*B) signaling pathway [44]. Accordingly, we examined whether BCP suppresses the LPS-induced inflamm-aging response in RAW264.7 cells through effects on the NF-*κ*B signaling pathway. NF-*κ*B is an important transcription factor ubiquitous in the central nervous system and a key transcription factor involved in oxidative stress and the inflammatory response [45]. The NF-*κ*B signaling pathway is involved in inhibition of oxidative stress-induced cell damage [46]. p65 and I-*κ*B*α* have been identified as indicators for detection of the NF-*κ*B signaling pathway. I-*κ*B*α*, an NF-*κ*B inhibitor, enters the nucleus to cause separation of NF-*κ*B from DNA and inhibits the NF-*κ*B signaling pathway. At the inactive stage, p65 binds I-*κ*B protein to form a p65-p50-I-*κ*B*α* complex that localizes in the cytoplasm, leading to accumulation of I-*κ*B*α*. Upon stimulation by inflammatory factors, NF-*κ*B-induced mitogen protein kinase is activated, following which p50 and p65 enter the nucleus to regulate the transcription of target genes. Consequently, I-*κκ* is degraded while levels of I-*κ*B*α* and phosphorylated I-*κ*B*α* are increased [47]. To examine our hypothesis, expression levels of p65 and I-*κ*B*α* and their phosphorylated counterparts were examined. After LPS stimulation, phosphorylated p65 and I-*κ*B*α* proteins were upregulated while I-*κ*B*α* protein was downregulated. Conversely, after treatment with BCP, phosphorylated p65 and I-*κ*B*α* proteins were downregulated while I-*κ*B*α* protein was upregulated, supporting the theory that BCP inhibits the occurrence of inflamm-aging through the NF-*κ*B signaling pathway. In addition, activation of Toll-like receptors (TLR), a transmembrane protein, can trigger binding to myeloid differentiation factor 88 (MyD88) to activate interleukin-1 receptor-associated kinase, and finally, the NF-*κ*B signaling pathway through I-*κ*B kinase, followed by stimulation of transcription factor activity and expression of a series of inflammatory factors to mediate the inflammatory response. TLR2, TLR4, and MyD88 are considered key proteins of the NF-*κ*B signaling pathway [48]. While BCP was shown to inhibit the occurrence of inflamm-aging through the NF-*κ*B signaling pathway in this study, it remains to be established whether BCP-mediated modulation of NF-*κ*B signaling occurs through regulation of TLR2, TLR4, and MyD88 proteins.

Mitochondria serve as the “energy factory” of cells, and their dysfunction triggers ROS production [49, 50]. Previous reports suggest the loss of mitochondrial membrane potential leads to aging and inflammation of cells [51]. In our experiments, LPS significantly induced ROS generation and decreased mitochondrial membrane potential in RAW264.7 cells. These effects of LPS were effectively reversed by BCP.

Breakdown of lysosomes leads to release of SA-*β*-gal and ultimately an increase in its content in aging cells [52]. SAHF, originally identified by Narita and coworkers in senescent cells, can be utilized as a specific aging index [53, 54]. In normal cells, p53 combines with ubiquitinase and is degraded through the ubiquitination pathway. Upon stimulation of cells, phosphorylated p53 can block the effects of the p53-ubiquitinase combination to further induce aging due to cell cycle arrest [55]. p16, a cell cycle regulatory protein, forms a complex with CDK4 or CDK6 to block cell cycle progression. Phosphorylated p16 sequesters the cyclin D-dependent kinases, CDK4, and CDK6, causing G1 phase cell cycle arrest and senescence [56, 57]. Consistent with these theories, the number of SA-*β*-gal-positive macrophage cells and SAHF phenomenon were increased significantly after LPS stimulation in our experiments. Moreover, protein levels of p53, p16, and phosphorylated p53 and p16 were elevated in LPS-stimulated macrophages. Notably, BCP inhibit both SA-*β*-gal expression and SAHF as well as levels of p53, p16, and their phosphorylated proteins in LPS-stimulated macrophages.

BCP used in the current study was obtained previously in our laboratory. BCP (Mw = 29 kDa) contains a backbone of (1 ⟶ 5)-linked Ara, (1 ⟶ 4)-linked Gal, and (1 ⟶ 3)-linked Gal residues with occasional branches at O-6 composed of (1 ⟶ 4)-linked Glc and terminated with Gal residues [27]. *Lycium ruthenicum* polysaccharide, mainly composed of arabinose, galactose, and rhamnose, is reported to significantly inhibit the production of NO, TNF-*α* and IL-1*β*, and expression of iNOS induced by LPS to regulate TLR4 and NF-*κ*B signaling pathways, leading to inhibition of inflammations [44]. Moreover, *Ganoderma lucidum* polysaccharide, which is mainly composed of glucose, inhibits the binding of L-selectin-sTyr/sLeX, lymphocyte homing and activation of the complement system to suppress complement and cytokine-mediated inflammatory pathways, inducing strong anti-inflammatory effects [58]. The above monosaccharide components are possibly implicated in the anti-inflamm-aging effect of BCP. Further research is required to establish the effective components and optimal proportions of monosaccharides that exert the most favorable therapeutic effects.

## 5. Conclusion

BCP significantly inhibits LPS-stimulated inflamm-aging of macrophages through the NF-*κ*B signaling pathway. This study provides a theoretical basis for further research and development of anti-inflamm-aging drugs containing BCP.

## Data Availability

The data used to support the findings of this study are available from the corresponding author upon request.
